# Supplementary material for: An open-source framework for large-scale, flexible evaluation of biomedical text mining systems
Source: J Biomed Discov Collab. 2008 Jan 29;3:1. doi: 10.1186/1747-5333-3-1 (PMC2276192; doi:10.1186/1747-5333-3-1)
Supplement: Additional file 1 — Evaluation platform source code. The additional file contains the software evaluation framework discussed in this paper. The most current release can be downloaded from SourceForge.net [1]. See the accompanying README file for instructions on installation and use. Also included in the distribution is a collection of UIMA wrappers for some commonly used BioNLP tools and annotated corpora. [file 1747-5333-3-1-S1.zip › bionlp_uima_repository-1.0.1/docs/api/src-html/edu/uchsc/ccp/uima/annotation/CCPAnnotator_Type.html]

```
001    
002    /* First created by JCasGen Tue Aug 28 16:44:10 MDT 2007 */
003    package edu.uchsc.ccp.uima.annotation;
004    
005    import org.apache.uima.cas.Feature;
006    import org.apache.uima.cas.FeatureStructure;
007    import org.apache.uima.cas.Type;
008    import org.apache.uima.cas.impl.CASImpl;
009    import org.apache.uima.cas.impl.FSGenerator;
010    import org.apache.uima.cas.impl.FeatureImpl;
011    import org.apache.uima.cas.impl.TypeImpl;
012    import org.apache.uima.jcas.JCas;
013    import org.apache.uima.jcas.JCasRegistry;
014    import org.apache.uima.jcas.cas.TOP_Type;
015    
016    /** The annotator object contains information which is used to determine who/what generated an annotation.
017     * Updated by JCasGen Tue Aug 28 16:44:10 MDT 2007
018     * @generated */
019    public class CCPAnnotator_Type extends TOP_Type {
020      /** @generated */
021      protected FSGenerator getFSGenerator() {return fsGenerator;}
022      /** @generated */
023      private final FSGenerator fsGenerator = 
024        new FSGenerator() {
025          public FeatureStructure createFS(int addr, CASImpl cas) {
026                             if (CCPAnnotator_Type.this.useExistingInstance) {
027                               // Return eq fs instance if already created
028                         FeatureStructure fs = CCPAnnotator_Type.this.jcas.getJfsFromCaddr(addr);
029                         if (null == fs) {
030                           fs = new CCPAnnotator(addr, CCPAnnotator_Type.this);
031                               CCPAnnotator_Type.this.jcas.putJfsFromCaddr(addr, fs);
032                               return fs;
033                         }
034                         return fs;
035            } else return new CCPAnnotator(addr, CCPAnnotator_Type.this);
036              }
037        };
038      /** @generated */
039      public final static int typeIndexID = CCPAnnotator.typeIndexID;
040      /** @generated 
041         @modifiable */
042      public final static boolean featOkTst = JCasRegistry.getFeatOkTst("edu.uchsc.ccp.uima.annotation.CCPAnnotator");
043     
044      /** @generated */
045      final Feature casFeat_annotatorID;
046      /** @generated */
047      final int     casFeatCode_annotatorID;
048      /** @generated */ 
049      public int getAnnotatorID(int addr) {
050            if (featOkTst && casFeat_annotatorID == null)
051          jcas.throwFeatMissing("annotatorID", "edu.uchsc.ccp.uima.annotation.CCPAnnotator");
052        return ll_cas.ll_getIntValue(addr, casFeatCode_annotatorID);
053      }
054      /** @generated */    
055      public void setAnnotatorID(int addr, int v) {
056            if (featOkTst && casFeat_annotatorID == null)
057          jcas.throwFeatMissing("annotatorID", "edu.uchsc.ccp.uima.annotation.CCPAnnotator");
058        ll_cas.ll_setIntValue(addr, casFeatCode_annotatorID, v);}
059        
060      
061     
062      /** @generated */
063      final Feature casFeat_firstName;
064      /** @generated */
065      final int     casFeatCode_firstName;
066      /** @generated */ 
067      public String getFirstName(int addr) {
068            if (featOkTst && casFeat_firstName == null)
069          jcas.throwFeatMissing("firstName", "edu.uchsc.ccp.uima.annotation.CCPAnnotator");
070        return ll_cas.ll_getStringValue(addr, casFeatCode_firstName);
071      }
072      /** @generated */    
073      public void setFirstName(int addr, String v) {
074            if (featOkTst && casFeat_firstName == null)
075          jcas.throwFeatMissing("firstName", "edu.uchsc.ccp.uima.annotation.CCPAnnotator");
076        ll_cas.ll_setStringValue(addr, casFeatCode_firstName, v);}
077        
078      
079     
080      /** @generated */
081      final Feature casFeat_lastName;
082      /** @generated */
083      final int     casFeatCode_lastName;
084      /** @generated */ 
085      public String getLastName(int addr) {
086            if (featOkTst && casFeat_lastName == null)
087          jcas.throwFeatMissing("lastName", "edu.uchsc.ccp.uima.annotation.CCPAnnotator");
088        return ll_cas.ll_getStringValue(addr, casFeatCode_lastName);
089      }
090      /** @generated */    
091      public void setLastName(int addr, String v) {
092            if (featOkTst && casFeat_lastName == null)
093          jcas.throwFeatMissing("lastName", "edu.uchsc.ccp.uima.annotation.CCPAnnotator");
094        ll_cas.ll_setStringValue(addr, casFeatCode_lastName, v);}
095        
096      
097     
098      /** @generated */
099      final Feature casFeat_affiliation;
100      /** @generated */
101      final int     casFeatCode_affiliation;
102      /** @generated */ 
103      public String getAffiliation(int addr) {
104            if (featOkTst && casFeat_affiliation == null)
105          jcas.throwFeatMissing("affiliation", "edu.uchsc.ccp.uima.annotation.CCPAnnotator");
106        return ll_cas.ll_getStringValue(addr, casFeatCode_affiliation);
107      }
108      /** @generated */    
109      public void setAffiliation(int addr, String v) {
110            if (featOkTst && casFeat_affiliation == null)
111          jcas.throwFeatMissing("affiliation", "edu.uchsc.ccp.uima.annotation.CCPAnnotator");
112        ll_cas.ll_setStringValue(addr, casFeatCode_affiliation, v);}
113        
114      
115    
116    
117    
118      /** initialize variables to correspond with Cas Type and Features
119            * @generated */
120      public CCPAnnotator_Type(JCas jcas, Type casType) {
121        super(jcas, casType);
122        casImpl.getFSClassRegistry().addGeneratorForType((TypeImpl)this.casType, getFSGenerator());
123    
124     
125        casFeat_annotatorID = jcas.getRequiredFeatureDE(casType, "annotatorID", "uima.cas.Integer", featOkTst);
126        casFeatCode_annotatorID  = (null == casFeat_annotatorID) ? JCas.INVALID_FEATURE_CODE : ((FeatureImpl)casFeat_annotatorID).getCode();
127    
128     
129        casFeat_firstName = jcas.getRequiredFeatureDE(casType, "firstName", "uima.cas.String", featOkTst);
130        casFeatCode_firstName  = (null == casFeat_firstName) ? JCas.INVALID_FEATURE_CODE : ((FeatureImpl)casFeat_firstName).getCode();
131    
132     
133        casFeat_lastName = jcas.getRequiredFeatureDE(casType, "lastName", "uima.cas.String", featOkTst);
134        casFeatCode_lastName  = (null == casFeat_lastName) ? JCas.INVALID_FEATURE_CODE : ((FeatureImpl)casFeat_lastName).getCode();
135    
136     
137        casFeat_affiliation = jcas.getRequiredFeatureDE(casType, "affiliation", "uima.cas.String", featOkTst);
138        casFeatCode_affiliation  = (null == casFeat_affiliation) ? JCas.INVALID_FEATURE_CODE : ((FeatureImpl)casFeat_affiliation).getCode();
139    
140      }
141    }
142    
143    
144    
145
```
